# Optimal Filter Estimation for Lucas-Kanade Optical Flow

**DOI:** 10.3390/s120912694

**Published:** 2012-09-17

**Authors:** Nusrat Sharmin, Remus Brad

**Affiliations:** Computer Science Department, Lucian Blaga University of Sibiu, B-dul Victoriei 10, 550024 Sibiu, Romania; E-Mail: nusrat_nik@yahoo.com

**Keywords:** optical flow, Lucas-Kanade, Gaussian filtering, optimal filtering

## Abstract

Optical flow algorithms offer a way to estimate motion from a sequence of images. The computation of optical flow plays a key-role in several computer vision applications, including motion detection and segmentation, frame interpolation, three-dimensional scene reconstruction, robot navigation and video compression. In the case of gradient based optical flow implementation, the pre-filtering step plays a vital role, not only for accurate computation of optical flow, but also for the improvement of performance. Generally, in optical flow computation, filtering is used at the initial level on original input images and afterwards, the images are resized. In this paper, we propose an image filtering approach as a pre-processing step for the Lucas-Kanade pyramidal optical flow algorithm. Based on a study of different types of filtering methods and applied on the Iterative Refined Lucas-Kanade, we have concluded on the best filtering practice. As the Gaussian smoothing filter was selected, an empirical approach for the Gaussian variance estimation was introduced. Tested on the Middlebury image sequences, a correlation between the image intensity value and the standard deviation value of the Gaussian function was established. Finally, we have found that our selection method offers a better performance for the Lucas-Kanade optical flow algorithm.

## 1. Introduction

Unlike the processing of static images, much broader information can be extracted from time varying image sequences, this being one of the primary functions of a computer vision system. Obtaining motion information is a challenging task for machines, however, several techniques have been developed in order to obtain the requested motion field. By definition, the optical flow is the pattern of apparent motion of objects, surfaces and edges in a visual scene, caused by the relative motion between the observer and the scene.

In 1981, two differential-based optical flow algorithms were proposed, now considered as classics: one by Horn and Schunck [1] and the other by Lucas and Kanade [2]. Following Horn's definition, the motion field is the 2D projection of the 3D motion of surfaces in the world, whereas the optical flow is the apparent motion of the brightness patterns in the image. On the other hand, the Lucas-Kanade approach assumes that the flow is essentially constant in a local neighborhood of the pixel under consideration, and solves the basic optical flow equations for all the pixels in that neighborhood, by the least squares criterion. Many different optical flow algorithms have been developed since 1981, including extensions and modifications of the Horn-Schunck and Lucas-Kanade approaches. Black and Anandan [3] presented a robust estimation framework to deal with such outliers, but did not attempt to model the true statistics of brightness constancy errors and flow derivatives.

While introducing different optical flow methods, it was also necessary to evaluate the proposed methods. Barron, Fleet, and Beauchemin [4] provided a performance analysis of a number of optical flow techniques, which emphasizes on the accuracy and density of measurements.

In 2000, Christmas [5] introduced a filtering requirement for the computation of gradient-based optical flow. Also, different authors recommended the use of a filtering method, such as Fleet and Langley [6] and Xiao *et al.* [7]. In most cases, the authors employed one filtering method in the evaluation of optical flow. In [7] a multi-cue driven adaptive bilateral filter was proposed in order to regularize the flow computation, which was able to achieve a smooth optical flow field with highly desirable motion discontinuities. According to Fleet *et al.* [6], applying a simple recursive filter is necessary to achieve temporal smoothing and to compute the 2D flow from component velocity constraints using a spatio-temporal least square minimization. Nevertheless, the importance of filtering techniques in obtaining an accurate optical flow is emphasized in [8].

The design of optimal spatio-temporal filters, especially the ones proposed by Simoncelli [9] is extensively presented in [10], along with the use of 2D Gaussian as pre-processing. Only two values for the Gaussian standard deviation have been investigated, as the combination with other 3D filters provided an improvement of optical flow detection. The same approach of combining the spatio-temporal filters of Baron and Simoncelli and optimal in the aim of reducing the motion estimation error is presented by Elad *et al.* in [11]. In order to measure the concentration field of an injected gaseous fuel, Iffa *et al.* [12] employ a pyramidal Lucas-Kanade flow determination in conjunction with a 5 × 5 kernel Gaussian filter.

In this paper, we focused on improving the accuracy of optical flow estimation by using the appropriate filtering method. As image filtering is essential in many applications, including smoothing, noise removal or edge detection, in the case of optical flow, we have investigated the filtering technique as a required preprocessing step. Also, in Section 3 we have analyzed different filtering methods in order to select the most suitable one. Section 4 presents a novel method for the selection of the appropriate Gaussian filter parameter, as discussed and sumarized in Section 5.

## 2. The Lukas-Kanade Optical Flow and Coarse-to-Fine Approach

We focused our investigation on the Lucas-Kanade optical flow determination. This gradient-based approach uses the constraint of pixel intensities constancy:

(1) I(x,y,t)=I(x+dx,y+dy,t+dt)

**Figure 1.**
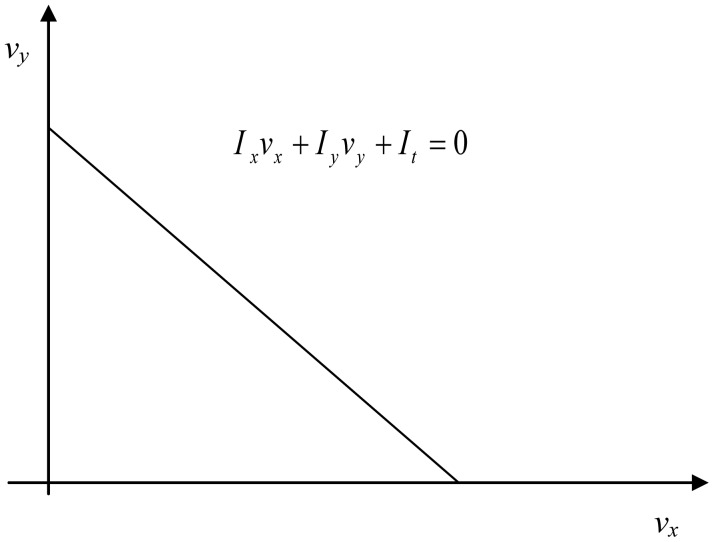
Optical flow constraint line.

The optical flow constraint equation derived from the Taylor expansion of Equation (1) was introduced by Horn and Schunk in [1]. Having two unknown variables in one equation, it gives the aperture problem:

(2) $$I_{x}v_{x}+I_{y}v_{y}+I_{t}=0$$

where *I_x_*, *I_y_* and *I_t_* denote the derivatives of the image function *I*(*x, y*) with respect to *x*, *y* and *t* (see Figure 1). The vector *V* = (*v_x_*, *v_y_*) defines the velocity vector in *x* and *y* direction.

**Figure 2.**
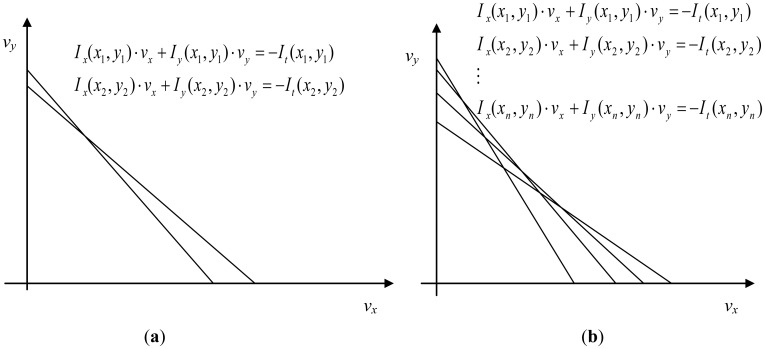
Intersection of (**a**) two optical flow and (**b**) several optical flow constraint lines.

This problem cannot be solved as there are two unknowns in one equation, but if a small region is supposed to have the same velocity, the problem has a solution. Thus, *V* can be found at the intersection of the Horn-Schunk constraints for each pixel. If we consider only two pixels, we obtain one intersection point, as in Figure 2. According to Lucas-Kanade, usually, a region of several pixels is considered having the same velocity. The equations system is now over determined. Therefore, the least squared error solution is supposed to give a good estimation of the optic flow value for a pixel, as depicted in Figure 2(b).

The optical flow equation is assumed to be used for all pixels within a window centered on pixel *p*. Explicitly, the local flow vector (v_x_, *v_y_*) must satisfy the optical flow constraint for a region of pixels with the same velocity, expressed by:

(3) $$\begin{array}{l} I_{x}(x_{1},y_{1})\cdot v_{x}+I_{y}(x_{1},y_{1})\cdot v_{y}=-I_{t}(x_{1},y_{1})\\ I_{x}(x_{2},y_{2})\cdot v_{x}+I_{y}(x_{2},y_{2})\cdot v_{y}=-I_{t}(x_{2},y_{2})\\ \vdots \\ I_{x}(x_{n},y_{n})\cdot v_{x}+I_{y}(x_{n},y_{n})\cdot v_{y}=-I_{t}(x_{n},y_{n}) \end{array}$$

The equation system (3) can be rewritten using matrix-vector notation:

(4) $$\underset{A}{\underset{︸}{\left(\begin{array}{l} \begin{array}{cc} I_{x}(x_{1},y_{1}) & I_{y}(x_{1},y_{1}) \end{array}\\ \begin{array}{cc} I_{x}(x_{2},y_{2}) & I_{y}(x_{2},y_{2}) \end{array}\\ \vdots \\ \begin{array}{cc} I_{x}(x_{n},y_{n}) & I_{y}(x_{n},y_{n}) \end{array} \end{array}\right)\cdot }}\underset{v}{\underset{︸}{\left(\begin{array}{c} v_{x}\\ v_{y} \end{array}\right)}}=-\left(\underset{b}{\underset{︸}{\begin{array}{c} I_{t}(x_{1},y_{1})\\ I_{t}(x_{2},y_{2})\\ \vdots \\ I_{t}(x_{n},y_{n}) \end{array}}}\right)$$

This system has more equations than unknowns and thus it is usually over-determined. The Lucas-Kanade method obtains a compromise solution using the least square technique. In consequence, it solves the 2 × 2 system:

(5) $$\begin{array}{l} A^{T}Av=A^{T}b\\ or\\ v={\left(A^{T}A\right)}^{-1}A^{T}b \end{array}$$

**Figure 3.**
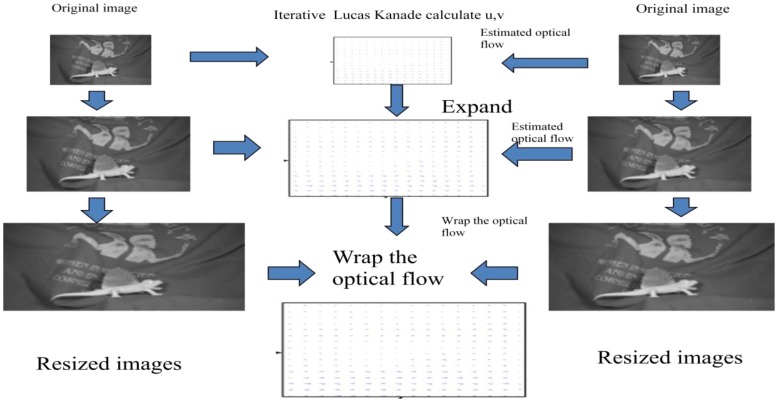
Coarse-to-fine optical flow estimation.

The Lucas-Kanade approach is a local optimization problem that cannot perform properly if the object movements are too large. As the gradient information is obtained by neighboring pixels, the real object motion cannot extend beyond the considered region. Also, the local neighborhood taken into account for the least squares approach is finite and there are few chances to correctly determine large movements. Therefore, it is common to use a pyramidal implementation. The input images are resized to a lower resolution, first by filtering with a low pass filter and then subsampled by a factor of 2, technique called coarse-to-fine approach, as shown in Figure 3. The computation of the optical flow is started with the lowest resolution images, at the highest pyramidal level. The result is passed then to the higher resolution level as an initial estimate. Running the algorithm on higher resolutions will cause higher accuracy for the flow field.

Bouguet describes in [13] an iterative implementation of the Lucas-Kanade method using a Gaussian pyramid. The Iterative Lucas-Kanade algorithm requires an estimate of the velocity for every pixel using the classical algorithm. Then, by means of a warping technique, the estimated flow will be warped on the image and the process is repeated until convergence.

## 3. An Empirical Method for Optimal Filter Selection

In this section, we present and discuss the results of our investigation. We have examined the performance of iterative Lucas-Kanade pyramidal optical flow algorithm together with different filtering techniques using well-known image sequences, provided with ground truth optical flow. The experimental were performed on a MATLAB R2010 platform using the standard available toolbox functions.

### 3.1. The Context of Evaluation

In the aim of experimental evaluation, we have employed the Middlebury dataset [14] which provides the ground truth. The testing set presents a variety of sequences, including hidden texture, realistic and complex scenes and non-rigid motion. For fair comparisons, we have used gray-scale images, two frame sequence and the brightness constancy assumption. Three data sets, such as “Dimetrodon”, “RubberWhale” and “Hydrangea” contains real world images with complex occlusions, while synthetic computer generated graphics are contained in four sets named “Grove2”, “Grove3”, “Urban2” and “Urban 3” [15]. The last set called “Venus” contains stereo images.

We have measured the performance of the estimated optical flow using both average angular error (AAE) and average endpoint error (AEE). The first error metric is the angle difference between the correct and estimated flow vectors defined by:

(6) $$\text{AAE}=\cos^{-1}(\hat{c}\cdot \hat{e})$$

where *ĉ* is the normalized correct motion vector and *ê* is the normalized estimate optical flow vector.

We have also evaluated the results by means of an absolute error, the flow endpoint error (EE) defined by:

(7) $$\text{AEE}=\sqrt{{\left(u-u_{GT}\right)}^{2}+{\left(v-v_{GT}\right)}^{2}}$$

where (*u*,*v*) is the estimated flow and (*u_GT_*, *v_GT_*) is the ground truth optical flow.

At the very first stage of our evaluation, we have used the Pyramidal Iterative Lucas-Kanade algorithm with three pyramidal levels, combined with different filtering techniques on the eight data sets of the Middlebury benchmark. For the preprocessing step of the optical flow estimation, eight different filtering techniques were employed, namely the Gaussian, Median, Mean, High Boost, Laplacian, LOG, Bilateral and Adaptive Noise Removal filtering. Bilateral filtering is a nonlinear filtering method first proposed by Tomasi *et al.* [16]. Although there are various applications, as reported by Paris *et al.* [17] and Elad [18], our idea was to smooth images while preserving their edges. The effects of several smoothing filters, togheter with the process of optimal parameters selection were presented by Malik *et al.* in [19].

In the case of the Gaussian filter, a standard deviation σ = 1 was used, the median filter had a 3 × 3 kernel, the LOG filter had a size of 5 × 5 and standard deviation σ = 0.5. The Mean filter had a 3 × 3 size, the Laplacian filter a value of alpha = 1 and for the case of Bilateral filter, a spatial-domain standard deviation of 0.1 and intensity-domain standard deviation of 0.1 were employed. We have also tested on all image sets, the “High Boost Filter” with 5 × 5 mask window and all pass factor weight ≥1 and the “Adaptive Noise Removal” filter that use neighborhoods of 3 × 3 to estimate the local image mean and standard deviation. The previous mentioned parameters were obtained after a large set of tests in which the parameters of each filter were varied and selected based on the minimum error (AAE and AEE). Thus, we have divided our experimental research into three sections, as presented in the following subsections.

### 3.2. Experimental Methodology

**Figure 4.**
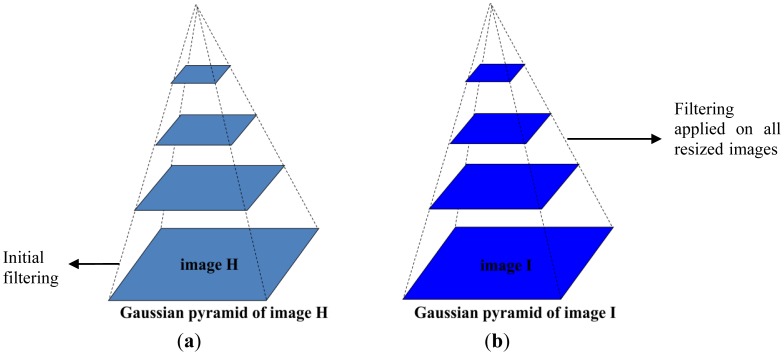
Coarse-to-fine optical flow estimation, (**a**) initial filtering and (**b**) filtering at all levels.

At the earlier stage of our investigation, the goal was to find the appropriate method for filtering in Lucas-Kanade optical flow estimation. Consequently, we have divided our experiment into three parts (Figure 4):

1. Filtering applied on input images for pyramidal optical flow computation
2. Filtering applied on all resized input images for pyramidal optical flow computation
3. Comparison between case 1 and 2

#### 3.2.1. Applying Filtering on Input Images at the Initial Level of Optical Flow Computation

**Figure 5.**
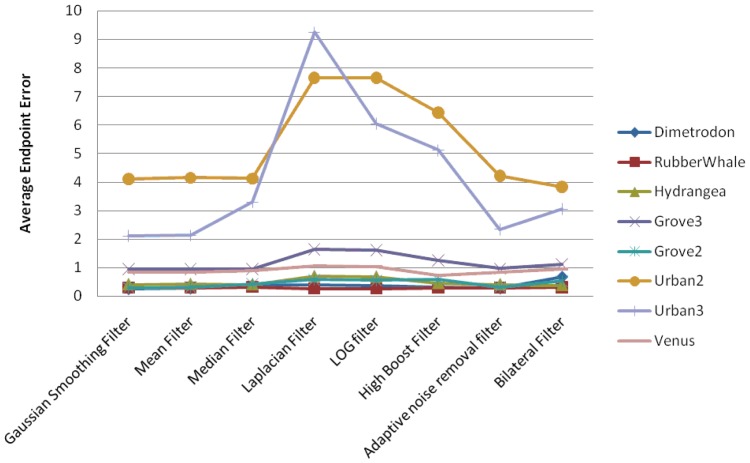
Average angular errors using different filters only on input images.

**Figure 6.**
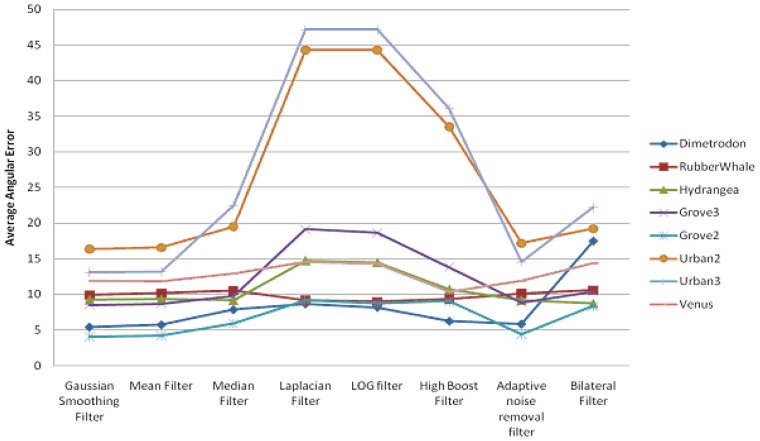
Average endpoint errors using different filters only on input images.

In this section, we present the experimental results for the pyramidal implementation of Lucas-Kanade, in the case where the filtering techniques have been applied before the optical flow estimation. We have concentrated our attention on the Average Angular Error and Average Endpoint Error estimated using eight different filtering techniques. We have found that AAE follows the same pattern as AEEs. Figure 5 shows a comparison between the AAEs (in degrees) computed on the Middlebury data sets, while the AEE variations are displayed in Figure 6. From the graphics depicted in Figure 6, one can observe the following:

- Gaussian, Mean, Median, Adaptive Noise Removal and Bilateral filtering result are comparatively better than the other one tested
- Smoothing filter increases significantly the accuracy of the detected flow field

Therefore, selecting the five best performing filters, we have varied the filters parameters in order to obtain the smallest AEE, as listed in Table 1.

Table 1 shows that the Gaussian Filter has the lowest error but for different standard deviations, depending on the input images. We have studied the correlation between the image statistics and the σ value in Section 4.

#### 3.2.2. Applying Filtering on All Images for the Pyramidal Optical Flow Computation

**Figure 7.**
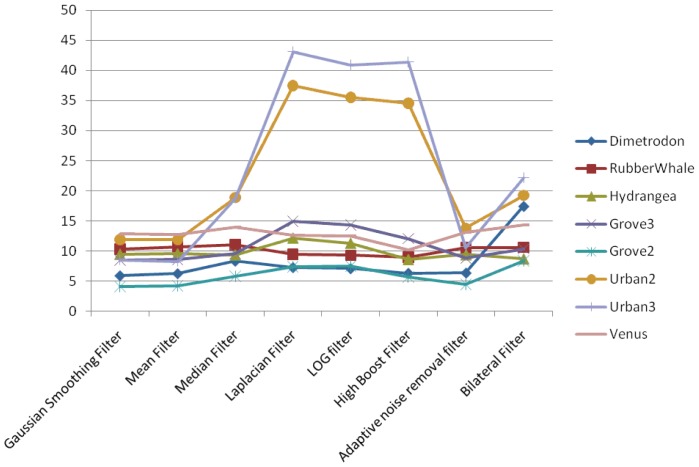
Average angular errors obtained using different filters on all resized images.

**Figure 8.**
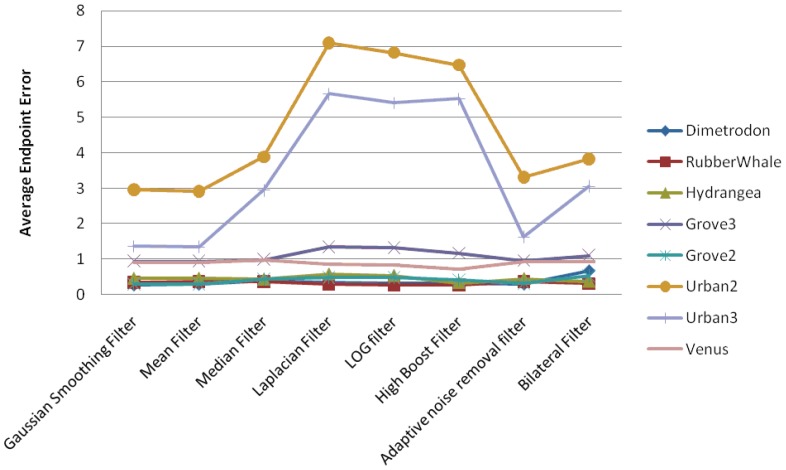
Average endpoint errors obtained using different filters on all resized images.

In the case of the pyramidal implementation of Lucas-Kanade, the input images are resized at each level to a lower resolution. Average angular error and average endpoint error are presented in Figures 7 and 8, respectively.

From the presented results and graphics, we can conclude that:

- Gaussian, Mean, Median, Adaptive Noise Removal and Bilateral filters result are comparatively better than the others
- Smoothing filters performs better than sharpening filters
- We recommended that for the Lucas-Kanade optical flow calculations it is better to use smoothing filters

In order to decide which method performs better, we made a checklist and an average ranking of the different filtering techniques. As a general conclusion from the experiments presented in Sections 3.2.1 and 3.2.2, in the case of pyramidal Lucas-Kanade optical flow, smoothing filters are recommended as the accuracy is improved. In order to decide which the best performing filter is and when it has to be applied, a comparison has been carried out, as shown in the following subsection.

#### 3.2.3. Comparison of Filtering Methods

As several filtering methods are considered, a checklist and an average ranking are presented in Tables 2 and 3 for selection of optimum filtering method.

Examining the values obtained in the above investigation, we can conclude that:

- filtering at all pyramidal levels is better than filtering only the initial images
- among all filtering methods, the Gaussian filter is optimal for computing Lucas-Kanade optical flow, as error is decreasing

**Figure 9.**
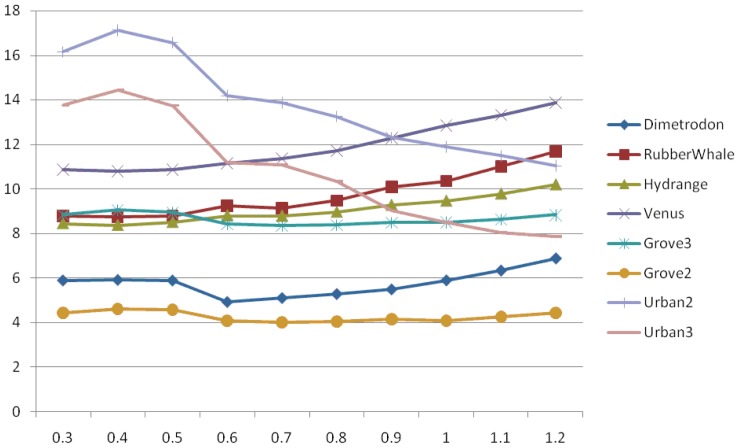
Average angular error for different σ values.

As the Gaussian filter performs better then any other considered filter, we have focused our investigation on finding the standard deviation parameter optimum value and drawing its dependency with error. Values presented in Figure 9 are for the case of a pyramidal Lucas-Kanade optical flow using Gaussian filter on all resized images.

## 4. A Novel Method for Estimating the Appropriate Gaussian Filtering Parameter

From the graphs in Figure 9, it is clearly shown that the appropriate σ value varies from image to image, but shows some common characteristic for the six image sets (Dimetrodon, RubberWhale, Hydrange, Grove3 and Grove2, Venus). The AAE increases with the increase of standard deviation, as for Urban2 and Urban3 image sequences it has a reverse behavior.

Therefore, we have tried to find the correlation between the image contents and the σ value, by computing a general measure, as the average intensity, for each image and for the entire dataset.

Based on several empirical tests and observations, we are proposing an algorithm for the estimation of the optimal filtering parameter:

- compute the mean intensity from input images
- find the reference point of Gaussian function
- using the values collected above, take a decision about the optimal parameter after a series of comparisons

The mean intensity of the test sequences was estimated using the values computed for two of the images and averageing of it at the end. For instance, in the case of the Dimetrodon image set, frame1 has an average image intensity of 0.3564, frame2 an average image intensity of 0.3567 and the average estimated intensity for the set was 0.3564. In Table 4, we have listed the estimated average intensity value of the Middlebury dataset.

Examining the plots in Figure 9, we have noticed the variation of the Gaussian function value according to standard deviation. In our investigation, we have employed a Gaussian function with the kernel defined on (−ksize/2, ksize/2), with a step of 2-ksize, where ksize = 6 × σ and a standard deviation of 1. In this case, the highest value of the Gaussian function was 0.3521, as shown in Table 5.

After the estimation of above mentioned values we have compared for each image set the reference point collected from the Gaussian function and the average image intensity. The standard deviation value of the Gaussian filter according to the image characteristics, was established after performing two types of comparisons. In the first case, we have just checked which value is greater than the other:

$$\begin{array}{ll} \text{If} & \left(\text{average image intensity}\geq \text{Highest Gaussian function value}\right)\\ & \text{Then choose standard deviation less than}1\\ \text{If} & \left(\text{average image intensity}<\text{highest Gaussian function Value}\right)\\ & \text{Then choose standard deviation higher than}1 \end{array}$$

Once completing this step for all image sets, we have obtained the results in Table 6. The bolded values are for image intensities greater than the reference point. Therefore, we can affirm that, if the average intensity value is equal or higher than the highest Gaussian function value, it is recommended to employ a standard deviation value of 1 or less than 1, and *vice versa*.

From Table 6, we have also noticed that the mean intensity value for the six image sets (Dimetrodon, RubberWhale, Hydrangea, Grove3, Grove2, and Venus) is greater than the reference value. Those are the sequences for which the AAE increase with the increase of σ value (see Figure 9). Therefore, it has been shown that, if we use small sigma values, the optical flow method will provide smaller errors. On the other hand, for Urban2 and Urban3, the mean intensity value is less than the reference point and comparing the two values, we have observed that it is better to use large σ values.

Based on several analyses, we have also suggested another method of choosing the standard deviation, as the ratio between the considered reference point and the image mean intensity value:

(8) ratio=highest Gaussian function value/mean intensity of input images

After computing the proposed ratio for all the benchmarks, the obtained values are presented in Table 7. For instance, in the case of Urban2 image set, the mean intensity was 0.21684, the reference point had a value of 0.3521, giving a ratio of 1.62.

Examining the obtained value, one can observe that its mean intensity is two times lower than the highest Gaussian value. Therefore, we have specified that the σ should be in the range of [1.62, 2]. As a generalization, the obtained ratio should be the lower bound for the optimal Gaussian filter parameter and the ceiling value of the ratio, the upper bound.

**Figure 10.**
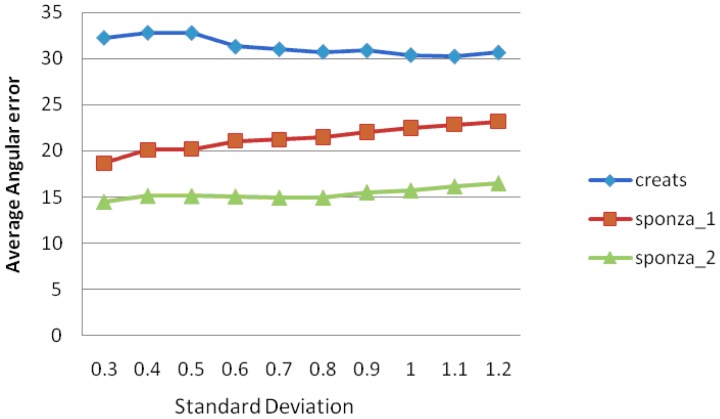
Average angular error measures of iterative pyramidal L-K optical flow by using Gaussian Filtering on all resized input images.

To confirm the above statement, we have tested three synthetic sets of images (http://visual.cs.ucl.ac.uk/pubs/algorithmSuitability/). Table 8 shows the mean intensity values employed for the selection of σ and Figure 10 the AAE measures for the pyramidal L-K optical flow, using Gaussian filtering on all resized input images.

## 5. Discussion and Conclusions

In this paper, we have presented an investigation on image filtering as a preprocessing level for the Lucas-Kanade optical flow computation framework. We have concluded not only on the fact that an optimal filtering must be performed at every pyramidal level, but also introduced a novel method for according the filter to the processed context. Also, from our study we have found that the Gaussian filter performs considerably better among different other filters.

Generally, in pyramidal optical flow computation, the input images are filtered only at the beginning and at the following levels, the images are being resized from that base. Our first experiment concerned the proper use of filtering, not only at the initial level, but subsequently at each pyramidal level. As several test sequences, together with the reference ground truth were available, an improvement of the error was obtained.

Since in our extensive research on the subject, we couldn't find any specifications regarding the optimal type of filter, we have considered the most referenced 2D ones, as Gaussian, Mean, Median, Bilateral, Adaptive Noise Removal or the High Boost filter. From the experimental results we have concluded that the Gaussian filtering is the most suitable in this regard, on the basis of computed average angular error and average endpoint error.

As the Gaussian filter was the most appropriate for pre-filtering the input images, we have investigated the relation between the standard deviation values of the Gaussian function and the image contents. From the plotted results, we have observed that there is no fixed σ value achieving the lowest error for any input sequence. Based on empirical observations, as the variation of error with standard deviation, we have established a correlation. Our novel method for selecting the σ value consists in observing the shape of the Gaussian function using a standard deviation of 1 and extracting the highest value. Comparing this reference point with the image average intensity can give an indication on the suitable value to be used. Also, we have found that the ratio between the image intensity and the highest Gaussian value can give an indication on the proper σ value. Finally, we concluded on the fact that computing the filter standard deviation from image characteristic offers a more accurate optical flow computation.

## Figures and Tables

**Table 1.** Lowest average endpoint error for best performing filters.

**Filter**	**Hidden texture**			**Synthetic**				**Stereo**

	**Dimetrodon**	**RubberWhale**	**Hydrangea**	**Grove3**	**Grove2**	**Urban2**	**Urban3**	**Venus**
Gaussian	4.7025	8.7112	8.3686	8.5187	3.9922	14.7064	11.5392	10.7046
smoothing σ	σ = 0.6	σ = 0.3	σ = 0.4	σ = 1	σ = 0.7	σ = 4.8	σ = 3.6	σ = 0.3
Mean	5.7210	10.143	9.3132	8.6578	4.2231	16.5652	13.1789	11.8439
Median	7.8426	10.5369	9.1439	9.7582	5.8926	19.5492	22.5063	12.9823
Adaptive Noise Removal	5.8196	10.1214	9.169	9.169	4.3921	17.1897	14.5003	11.9682
Bilateral σ = [0.1, 0.1]	17.4394	10.5945	8.716	10.3823	8.3447	19.2384	22.1881	14.3764

**Table 2.** Comparison between filtering methods at initial level and all levels on pyramidal Lucas-Kanade optical flow.

**Different filtering techniques**	**Hidden Texture**			**Synthetic**				**Stereo**

	**Dimetrdon**	**RubberWhale**	**Hydrangea**	**Grove3**	**Grove2**	**Urban2**	**Urban3**	**Venus**
Gaussian Smooth Filtering	x	X	x	-	-	-	-	-
Median Filtering	x	X	x	-	-	-	-	x
LOG filtering	-	X	-	-	-	-	-	x
Mean Filtering	x	X	x	-	-	-	-	-
High Boost Filtering	-	-	-	-	-	x	X	-
Laplacian Filtering	-	X	-	-	-	-	-	x
Adaptive Noise Removal filtering	x	X	x	-	-	-	-	x
Bilateral Filtering	-	-	-	-	-	-	-	-

Where x—recommended for initial filtering and X—recommended for all levels.

**Table 3.** Comparison between filtering methods at initial level and all pyramidal levels using average ranking.

**Filter**	**Filtering applied on input images at initial level (average ranking)**		**Filtering applied on all resized images at pyramidal levels (average ranking)**	

	**Average angular error (degrees)**	**Average endpoint error**	**Average angular error (degrees)**	**Average endpoint error**
Gaussian smooth	9.809825	1.156813	**8.9477**	**0.9484**
Median	12.2765	1.351225	**11.96125**	**1.307188**
LOG	20.6046875	2.27355	**17.29629**	**2.005838**
Mean	9.9558875	1.17025	**9.028563**	**0.947563**
High Boost	16.1461625	1.893188	**15.97896**	**1.903713**
Laplacian	20.8631375	2.688675	**18.05495**	**2.084288**
Adaptive Noise Removal	10.29116	1.141913	**9.64585**	**1.035**
Bilateral	13.909975	1.353625	**13.90998**	**1.353625**

**Table 4.** Average intensity value of Middlebury dataset.

**Image sets**	**Mean intensity value**
Dimetrodon	**0.3564**
RubberWhale	**0.52125**
Hydrangea	**0.4154**
Grove3	**0.39945**
Grove2	**0.3945**
Urban2	0.21684
Urban3	0.2504
Venus	**0.39645**

**Table 5.** Large values of Gauss function.

**x**	−3	−1.83	−0.6667	0.5	1.667	2.8333
**G(x)**	0.0044	0.0743	0.3194	0.3521	0.0995	0.0072

**Table 6.** Results of first comparison.

**Image sets**	**Mean intensity value**
Dimetrodon	**0.3564**
RubberWhale	**0.52125**
Hydrangea	**0.4154**
Grove3	**0.39945**
Grove2	**0.3945**
Urban2	0.21684
Urban3	0.2504
Venus	**0.39645**

**Table 7.** Result for the second comparison step.

**Image sets**	**Ratio**
Dimetrodon	**0.988**
RubberWhale	**0.6755**
Hydrangea	**0.8476**
Grove3	**0.8815**
Grove2	**0.8925**
Urban2	**1.6238**
Urban3	**1.4061**
Venus	**0.8881**

**Table 8.** Average of image intensity values.

**Image sets**	**Mean intensity value of each image sets**
Creats	0.4886
Sponza_1	0.33605
Sponaza_2	0.5741
